# Biochemical properties of a novel thermostable and highly xylose-tolerant β-xylosidase/α-arabinosidase from *Thermotoga thermarum*

**DOI:** 10.1186/1754-6834-6-27

**Published:** 2013-02-20

**Authors:** Hao Shi, Xun Li, Huaxiang Gu, Yu Zhang, Yingjuan Huang, Liangliang Wang, Fei Wang

**Affiliations:** 1 College of Chemical Engineering, Nanjing Forestry University, Nanjing, 210037, China; 2 Jiangsu Key Lab of Biomass-Based Green Fuels and Chemicals, Nanjing, 210037, China

**Keywords:** *Thermotoga thermarum*, β-xylosidase, α-arabinosidase, Xylose tolerant, Hemicellulose, Thermostability, Xylooligosaccharides

## Abstract

**Background:**

β-Xylosidase is an important constituent of the hemicellulase system and it plays an important role in hydrolyzing xylooligosaccharides to xylose. Xylose, a useful monose, has been utilized in a wide range of applications such as food, light, chemical as well as energy industry. Therefore, the xylose-tolerant β-xylosidase with high specific activity for bioconversion of xylooligosaccharides has a great potential in the fields as above.

**Results:**

A β-xylosidase gene (*Tth xynB3*) of 2,322 bp was cloned from the extremely thermophilic bacterium *Thermotoga thermarum* DSM 5069 that encodes a protein containing 774 amino acid residues, and was expressed in *Escherichia coli* BL21 (DE3). The phylogenetic trees of β-xylosidases were constructed using Neighbor-Joining (NJ) and Maximum-Parsimony (MP) methods. The phylogeny and amino acid analysis indicated that the Tth xynB3 β-xylosidase was a novel β-xylosidase of GH3. The optimal activity of the Tth xynB3 β-xylosidase was obtained at pH 6.0 and 95°C and was stable over a pH range of 5.0-7.5 and exhibited 2 h half-life at 85°C. The kinetic parameters *K*_*m*_ and *V*_*max*_ values for *p*-nitrophenyl-β-D-xylopyranoside and *p*-nitrophenyl-α-L-arabinofuranoside were 0.27 mM and 223.3 U/mg, 0.21 mM and 75 U/mg, respectively. The *k*_*cat*_/*K*_*m*_ values for *p*-nitrophenyl-β-D-xylopyranoside and *p*-nitrophenyl-α-L-arabinofuranoside were 1,173.4 mM^-1^ s^-1^ and 505.9 mM^-1^ s^-1^, respectively. It displayed high tolerance to xylose, with *K*_*i*_ value approximately 1000 mM. It was stimulated by xylose at higher concentration up to 500 mM, above which the enzyme activity of Tth xynB3 β-xylosidase was gradually decreased. However, it still remained approximately 50% of its original activity even if the concentration of xylose was as high as 1000 mM. It was also discovered that the Tth xynB3 β-xylosidase exhibited a high hydrolytic activity on xylooligosaccharides. When 5% substrate was incubated with 0.3 U Tth xynB3 β-xylosidase in 200 μL reaction system for 3 h, almost all the substrate was biodegraded into xylose.

**Conclusions:**

The article provides a useful and novel β-xylosidase displaying extraordinary and desirable properties: high xylose tolerance and catalytic activity at temperatures above 75°C, thermally stable and excellent hydrolytic activity on xylooligosaccharides.

## Background

Hemicellulose is the second most abundant renewable lignocellulosic biomass resource, in which xylan is a major component and it is mainly composed of a backbone of β-1,4-linked xylopyranosyl units with the presence of side groups’ substitution such as arabinosyl, acetyl and glucuronosyl [1-3]. The thorough degradation of xylan is a multi-step action and requires the synergistic action of several hydrolytic enzymes [4]. The enzymes primarily include xylanase (EC 3.2.1.8) and β-xylosidase (EC 3.2.1.37), which can hydrolyze xylan to yield xylooligosaccharides (XOs) and xyloses, respectively [5,6]. Moreover, additional enzymes such as α-L-arabinosidase, α-D-glucuronidase, and acetyl xylan esterase, can cleave the side-chain of glycosyl derivatives [6]. Ultimately hydrolysates of xylan, xylose and arabinose have been found to be useful for the applications in foods and fuel industries, as well as prebiotic exploitation [2,3]. In addition, bioethanol can be produced from lignocellulosic biomass using steam or aqueous ammonia pretreatment, followed by enzymatic hydrolysis and fermentation [7,8]. Xylanases can improve the hydrolysis of cellulose into fermentable sugars by depolymerizing xylans from the cellulose-hemicellulose compound, and furthermore enhance the access of cellulases to cellulose surfaces [8,9]. β-Xylosidases, displaying the similar function as xylanases, are important part of most microbial xylanolytic systems by attacking the non-reducing ends of XOs to release xylose or other oligosaccharides [10-13]. The catalytic process of β-xylosidases is considered as a double displacement mechanism requiring a glycosyl enzyme medium, like the catalysis of glycosidases [10]. Glycoside hydrolases, the most efficient enzymes presently known, split the glycosidic linkage between two carbohydrate residues [14]. It is well know that all the β-xylosidases are mainly divided into glycosyl hydrolase (GH) families 3, 39, 43, 52, and 54 based on their amino acid sequence similarities [15]. Typical substrate specificities, reaction mechanisms, and three-dimensional (3D) structures were reported in these members of each family [16]. However, a β-xylosidase from *Thermoanaerobacterium saccharolyticum* JW/SL-YS485 does not fit into any of the β-xylosidases families [17]. These β-xylosidases families together with all the other GH families are readily available on the continuously updated web site (http://www.cazy.org/Glycoside-Hydrolases.html) [18].

Although many β-xylosidases and their coding genes have been manipulated and characterized in plant, fungi, bacteria as well as archaea, few literatures about highly thermostable β-xylosidases are available in database. Indeed, enzyme with high thermostability is essential for the industrial application in biomass degradation, as it can prolong its service life and reduce the enzyme consumption [3]. Therefore, it serves as an efficient way in bioconversion for xylan degradation at high temperature.

*Thermotoga thermarum* isolated from continental solfataric springs at Lac Abbe (Djibouti, Africa), is an anaerobic hyperthermophile that grows at 80°C and at pH values ranging from 5.5 to 9.0, which has been reported to produce many hydrolases including β-xylosidase [19].

In this paper, we described the cloning, expression, purification and biochemical characterizations of Tth xynB3 β-xylosidase, the novel thermostable β-xylosidase from *T. thermarum*.

## Results

### Cloning and sequence analysis of Tth xynB3 β-xylosidase

Through the analysis of the genome sequence of*T. thermarum* DSM 5069, a protein (Theth_0138), defined as β-mannanase in Genbank, consists of a 2,322 bp fragment encoding 774 amino acids, which belongs to glycoside hydrolases family 3 (GH3). It shares the highest sequence similarity of 71% with the β-xylosidase from *Thermotoga maritima* MSB8 (Genbank No. NP_227892), and was revealed by whole-genome sequencing yet has not been biochemically characterized. Alignment of the Tth xynB3 β-xylosidase cluster with several representative members of GH3 indicated that they shared similar blocks. As we know, among all the members of GH3, aspartic acid acts as a catalytic nucleophile and glutamic acid as a catalytic proton donor. Based on present database, however, we are not able to obtain the three-dimensional (3D) structure and verify the role of the two active amino acids of the Tth xynB3 β-xylosidase. By the description of β-xylosidases of GH3, we know that the β-xylosidases has multi-domains, such as provisional β-D-glucoside glucohydrolase (PRK15098), β-glucosidase-related glycosidases (BglX), probable β-xylosidase (PLN03080) and GH3 C-terminal domain (pfam01915) [20]. Among these β-xylosidases from different GH families, the average length of amino acids sequence and multi-domains of each family are apparently different (http://www.ncbi.nlm.nih.gov/).

The results indicated that the protein (Theth_0138) could be a novel β-xylosidase (detailed data were described below). The DNA fragment of a protein (Theth_0138) gene was amplified from genomic DNA of *T. thermarum* DSM 5069, and ligated to pET-20b at *Nde I* and *Xho I* sites to generate plasmid pET-20b-*Tth xynB3*.

### Expression and purification of recombinant Tth xynB3 β-xylosidase

**Figure 1:**
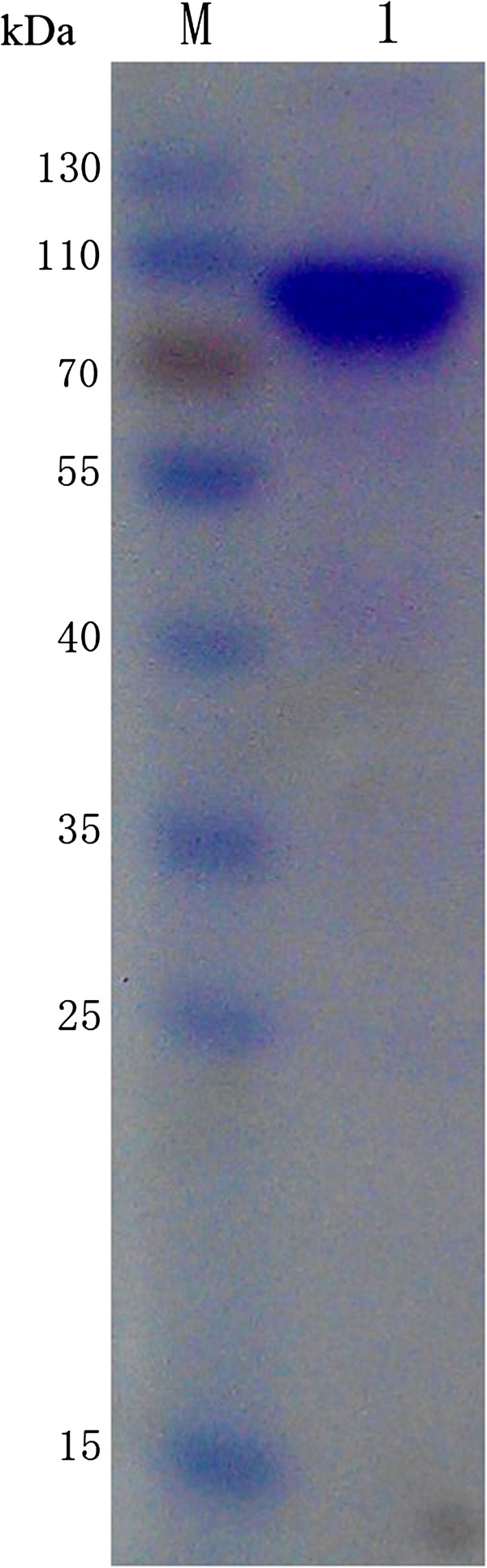
**SDS-PAGE analysis of recombinant Tth xynB3β-xylosidase in *E. coli *BL21 (DE3).** Lane M: protein marker, lane 1: purified Tth xynB3 β-xylosidase.

For functional analysis of the recombinant β-xylosidase, the plasmid pET-20b-*Tth xynB3* was expressed in *E. coli* BL21 (DE3). The heterologous protein was over-produced by inducing cells with 0.5 mM IPTG. The recombinant xylanase was purified through a heat treatment at 70°C for 30 min followed by a Ni-NTA affinity chromatography (Table 1). The extracts from the *E. coli* harboring the construct Tth xynB3 β-xylosidase displayed a single band at approximately 85 kD by SDS-PAGE analysis (Figure 1, lane 1), and the molecular weight (MW) of Tth xynB3 β-xylosidase conformed to the theoretical MW of the monomer (85,129 Da). Size exclusion chromatography was also performed using the Ӓ KTA*FPLC*™ system to determine the oligomerization state of the target protein. It was found that the native protein formed 5-mer in solution with a calculated MW 422, 474 Da according to the calibration curve of the gel filtration column. 

**Table 1:** **Purification of the recombinant Tth xynB3** β**-xylosidase**

**Purification step**	**Total volume (mL)**	**Total activity (U)**	**Total protein (mg)**	**Specific activity (U/mg)**	**Recovery (%)**	**Purification (fold)**
Crude extract^a^	10	2394	122	20	100	1
Heat treatment^b^	10	2155	39	55	90	2.8
Ni affinity chromatography^c^	1	2011	17	116	84	5.8

^a^ The recombinant strain was grown in LB medium (200 ml) with 1 μg ampicillin/ml at 37°C to OD_600_ 0.8 and was incubated further with isopropyl-β-thiogalactopyranoside (IPTG) for 12 h. The cells were harvested by centrifugation at 10,000 g for 15 min at 4°C and resuspended in 10 ml imidazole buffer (10 mL of 5 mM imidazole, 0.5 mM NaCl, and 20 mM Tris–HCl buffer, pH 7.9), followed by sonication.

^b^ The cell extracts after sonication were heat treated at 70°C for 30 min, and then cooled in an ice bath, centrifuged at 15,000 g for 20 min at 4°C and the supernatant was kept.

^c^ The obtained supernatants were loaded on to an immobilized metal affinity column (Novagen, USA), and eluted with 0.4 M imidazole, 0.5 M NaCl, and 20 mM Tris–HCl buffer (pH 7.9).

### Biochemical properties of Tth xynB3 β-xylosidase

**Figure 2:**
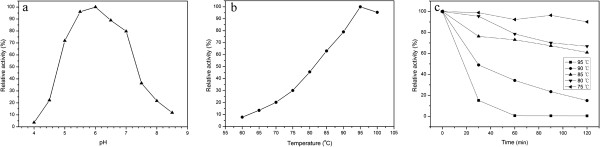
**Effects of pH and temperature on the activity and stability of the recombinant Tth xynB3β-xylosidase. a**) Effect of pH on Tth xynB3 β-xylosidase activity. **b**) Effect of temperature on Tth xynB3 β-xylosidase activity. **c**) The thermostability of the Tth xynB3 β-xylosidase. The residual activity was monitored while the enzyme was incubated at 75°C (*filled* left *triangles*), 80°C (*filled down triangles*), 85°C (*filled up triangles*), 90°C (*filled circles*) and 95°C (*filled squares*). The maximum activity was defined as 100% (**a**, **b**) or initial activity was defined as 100% (**c**).

The Tth xynB3 β-xylosidase exhibited the highest enzyme activity at pH 6.0, while its relative activity all remained high, approximately 70% of the maximum activity, with the pH ranging from 5.0 to 7.0 (Figure 2a). The β-xylosidase exhibited its optimal activity at 95°C (Figure 2b), and it retained more than 50% of its initial activity at 75°C-85°C for 2 h when tested at pH 6.0 (Figure 2c), and as indicated the half-life of the recombinant β-xylosidase was approximately 2 h at 85°C. 

The effects of cations and chemical reagents on the enzyme activity were also investigated, and the results were shown in Table 2. In various assays, the enzyme activity was significantly influenced by 1 mM concentration of Cu^2+^, Zn^2+^, Al^3+^, Mn^2+^ and Co^2+^ and 10 mM concentration of Ni^2+^, Zn^2+^, Mn^2+^, Ba^2+^ and EDTA. In addition, 0.05% Tween 60 and Tris also significantly affected the enzyme activity. The results of biochemical properties for α-arabinosidase were almost the same as those of the β-xylosidase (data was not detailed in this paper). 

**Table 2:** **Effects of cations and chemical reagents on purified Tth xynB3** β**-xylosidase activity**

**Cations**^**a**^	**Residual activity (%)**
Control	100
Mg^2+^	102/99
Zn^2+^	85/33
Mn^2+^	107/117
Ba^2+^	99/187
Ca^2+^	99/96
Al^3+^	87/ND^c^
Cu^2+^	4/2
Co^2+^	89/ND
Ni^2+^	100/63
Chemical reagents^b^	
EDTA	103/114
Tween 60	105/ND
Tris	107/ND
SDS	98/ ND

^a^ Final concentration, the former value in the table was determined at 1 mM and the latter was determined at 10 mM. ^b^ Final concentration, the values in the table were determined at 1 mM (or 10 mM, the latter values), 0.05%, 0.05% and 0.1% for EDTA, Tween 60, Tris, and SDS, respectively. ^c^ ND: not determined. Values shown were the means of duplicate experiments, and the variations about the means were below 5%.

### Effect of xylose on Tth xynB3 β-xylosidase activity and substrate specificity

**Figure 3:**
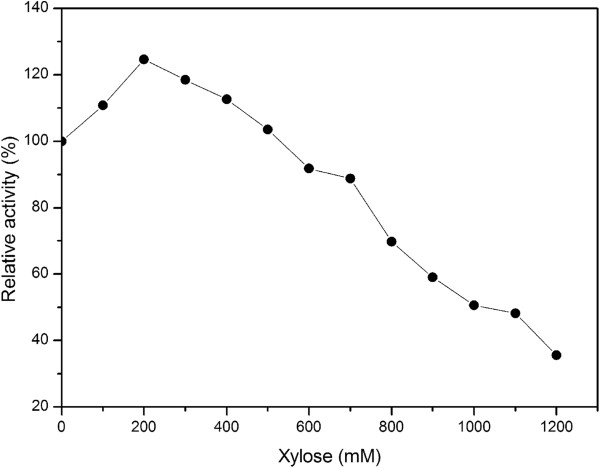
**Effects of xylose on Tth xynB3β-xylosidase activity.** The reaction was conducted with *p*-nitrophenyl-β-D-xylopyranoside as the substrate. The values were the mean of three separate experiments, and the variations about the mean were all below 5%.

The enzyme was able to hydrolyze *p*-nitrophenyl-β-D-xylopyranoside (*p*NPX) and *p*-nitrophenyl-α-L-arabinofuranoside (*p*NPAF), and almost no other glycosidase activity was detected over *p*-nitrophenyl-β-D-glucopyranoside, *p*-nitrophenyl α-D-glucopyranoside, caboxy methyl cellulose (CMC), linear arabinan and sucrose. The dependence of the enzymatic reaction rate on the substrates concentration followed Michaelis-Menten kinetics, with the kinetic parameters *K*_*m*_ and *V*_*max*_ values of 0.27 mM and 223.3 U/mg for *p*NPX, 0.21 mM and 75.0 U/mg for pNPAF under optimal conditions. The *k*_*cat*_/*K*_*m*_ value for *p*-nitrophenyl-α-L-arabinofuranoside was 505.9 mM^-1^ s^-1^. The *k*_*cat*_/*K*_*m*_ value of 1173.4 mM^-1^ s^-1^ for *p*NPX was significantly higher than that of β-xylosidase from *Aspergillus awamori*[15]. However, the turnover number *k*_*cat*_ for *p*NPX was 3.1-fold than that of *p*NPAF, and the catalytic efficiency constant *k*_*cat*_/*K*_*m*_ was 2.3-fold than that of *p*NPAF. The activity of Tth xynB3 β-xylosidase was stimulated by xylose at concentrations up to 500 mM. In the presence of 200 mM xylose, enzyme activity increased to a maximum value with 20% more than that of the control without xylose (Figure 3). With further increase of the xylose, the enzyme activity of Tth xynB3 β-xylosidase was gradually inhibited, with a *K*_*i*_ of 1000 mM xylose (Figure 3). The enzymatic characteristics of the xylose-tolerant β-xylosidase from other microorganisms were summarized in Table 3. This implies that these enzymes possess many distinct features, especially in their catalytic properties. 

**Table 3:** **Characteristics of highly xylose-tolerant** β**-xylosidase from *T. thermarum *DSM 5069 and other microorganisms**

**Strain**	***K***_***m***_**(mM)**		***V***_***max***_**(U/mg)**		***K***_***i***_**for xylose (mM)**	***k***_***cat***_***/K***_***m***_**(mM**^**-1**^**s**^**-1**^**)**		**Optimal temp (°C)**
	^**a**^***p*NPX**	^**b**^***p*NPAF**	***p*NPX**	***p*NPAF**		***p*NPX**	***p*NPAF**	
*T. thermarum*	0.27	0.21	223.2	75.0	1000	1173.4	505.9	95
^d^ Unnamed bacterium [26]	3.43	2.23	^c^ND	ND	76.0	8.1	2.5	40
*Paecilomyces thermophila*[22]	4.3	ND	ND	ND	139	ND	ND	55
*Scytalidium thermophilum*[21]	1.3	ND	88	ND	^e^ < 600	ND	ND	60

^a^*p*NPX: *p*-nitrophenyl-β-D-glucopyranoside.

^b^*p*NPAF: *p*-nitrophenyl-α-L-arabinofuranoside.

^c^ ND: not determined.

^d^ Isolated from yak rumen.

^e^ Calculated by the data based on the reference [21].

### Xylooligosaccharides degradation of Tth xynB3 β-xylosidase

**Figure 4:**
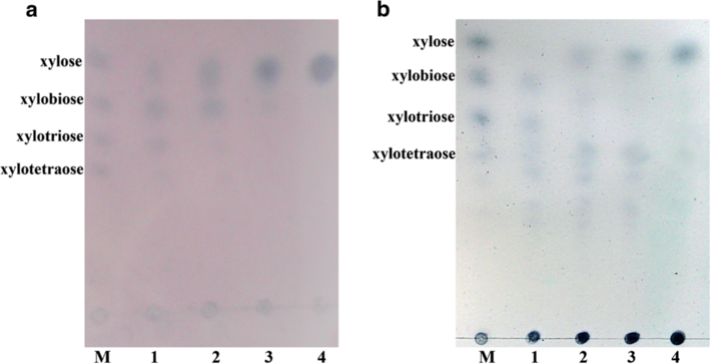
**Analysis of xylooligosaccharides hydrolyzed by Tth xynB3** β**-xylosidase.** The products of the reaction were determined using thin layer chromatography. M, mixture of xylose, xylobiose, xylotriose and xylotetraose (2.5% each, wt/vol). **a** Lane 1, 2, 3, 4: samples of xylobiose, xylotriose and xylotetraose (5%, wt/vol) incubated with Tth xynB3 β-xylosidase (0.3 U) for 0.5h, 1 h, 2 h, 3h, respectively. **b** Lane 1: samples of XOs obtained from cornstalk without hydrolysis using Tth xynB3 β-xylosidase, lane 2, 3, 4: samples of XOs obtained from cornstalk incubated with Tth xynB3 β-xylosidase (0.3 U) for 1 h, 2 h, 3h, respectively.

Production of xyloses by the purified Tth xynB3 β-xylosidase was examined using the thin layer chromatography (TLC) (Figure 4). The xyloses were generated from 10% XOs (isopyknic xylobiose, xylotriose and xylotetraose respectively) or from the hydrolysis of cornstalk by xylanse. After the hydrolysis for 3 h, the XOs from both sources were found to be biodegraded into xylose completely, and the final concentration of xylose in the reaction reached at approximately 360 mM. 

### Phylogenies analysis of Tth xynB3 β-xylosidase

**Figure 5:**
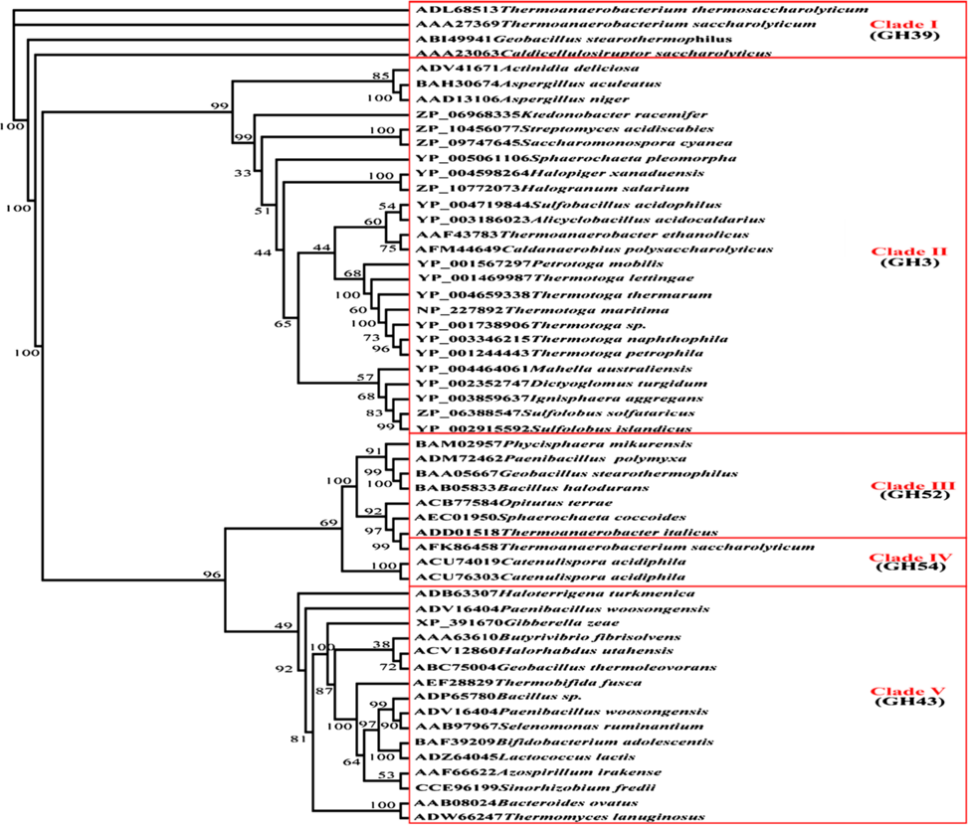
**Maximum-Parsimony (MP) tree results from analysis of Tth xynB3β-xylosidases of 55 amino acid sequences.** Numbers on nodes correspond to percentage bootstrap values for 1000 replicates.

To gain deeper insight into the evolutionary relationship among β-xylosidases, the phylogenetic trees generated from 55 candidate sequences were constructed using the NJ method and the MP method separately; both supported almost the same topological structures (NJ tree was not shown). The phylogenetic trees revealed the presence of five well-supported clades and each clade consisted of a separated monophyletic group. Clade I was the GH39 β-xylosidases from bacteria, Clade II was the GH3 β-xylosidases from bacteria, archaea and fungi, Clade III was the GH52 β-xylosidases from bacteria, Clade IV was the GH54 β-xylosidases from bacteria and Clade V was the GH43 β-xylosidases from bacteria and archaea. Among these families, the members of β-xylosidases in GH39, GH52 and GH54 were all from bacteria, and almost no information was available in fungi and archaea. However, other β-xylosidases in GH3 and GH43 were widely distributed. Clade II mainly contained mesophilic strains, thermophiles and hyperthermophiles. From the phylogenetic tree it was exhibited that there were several subclades in Clade II, among which the members of hyperthermophilic genus *Thermotoga* had a close relationship with *Petrotoga mobilis* and other hyperthermophiles, and *T. thermarum* Tth xynB3 β-xylosidase clustered together with the same genus *Thermotoga* β-xylosidases (Figure 5). Located at the boundary of the genus *Thermotoga*, β-xylosidases from *T. thermarum* and *T. lettingae* shared apparently distant relationship with the *T. petrophila* β-xylosidase. Therefore, it was postulated that their biochemical properties might be different. 

## Discussions

Based on amino acid sequence similarities, 130 families were found in Glycoside Hydrolases, among which GH 3, 39, 43, 52, and 54 contained β-xylosidases [15]. Through the blast at GenBank, the amino acid sequence analysis indicated that Tth xynB3 β-xylosidase belonged to GH3, and it shared the highest sequence similarity of 71% with the β-xylosidase from *Thermotoga sp.* (ZP_10919422) and the *Thermotoga maritima* (NP_227892). Moreover, it also shared the 71% similarity with the putative β-mannanase from the *Thermotoga neapolitana* (YP_002534158). So far as we know, the amino acid sequences of Tth xynB3 β-xylosidase was described as a β-mannanase in NCBI database. However, it has been confirmed as a β-xylosidase in details as described above. In most cases, two glutamic acid residues, or aspartic acid and glutamic acid residues are the catalytic nucleophile and proton donor in glycosyl hydrolases. However, the optimal template on database for homology modeling is 2X41A, which only shared 30% similarity with the Tth xynB3 β-xylosidase. Thus, no 3D structure of the Tth xynB3 β-xylosidase was obtained. Furthermore, it’s hard to distinguish which two amino acids are the catalytic nucleophile and proton donor in Tth xynB3 β-xylosidase.

This is the first report on the purification and characterization of a Tth xynB3 β-xylosidase from *T. thermarum*. The Phylogenies analysis and enzymatic properties showed that the Tth xynB3 β-xylosidase was distant with the xylose-tolerant β-xylosidase from *Paecilomyces thermophila* and *Scytalidium thermophilum*[21,22] (Table 3). The phylogenetic tree also revealed a close relationship between *T. thermarum* β-xylosidase and *T. maritima* β*-*xylosidase. *T.maritima * β-xylosidase has been described as a hyperthermophilic β-xylosidase with high thermostability at temperature 80°C [23]. Other β-xylosidases of genus *Thermotoga* including *T. thermarum* β-xylosidase have not been studied yet. As 71% amino acid sequences similarity was found between *T. thermarum* β-xylosidase and *T. maritime* β-xylosidase, it was inferred that the Tth xynB3 β-xylosidase could be a novel hyperthermophilic β-xylosidase with some specific properties.

Lignocellulosic biomass includes approximately 70% cellulose and 30% xylan [24]. Enhancing the biodegradation performance of hemicellulases on lignocellulosic biomass is of considerable significance for biorefinery [25]. Hemicellulose, which is mainly composed of xylan, a ubiquitous component of plants, consists of polysaccharides, each reflecting polydispersity, polymolecularity, and polydiversity. β-xylosidase is known to be the key enzyme for converting XOs to xylose which is the main end-product of xylan [22]. To our knowledge, xylose is a strong inhibitor of β-xylosidases. Therefore, β-xylosidases with high tolerance for xylose have great potential in conversion of hemicellulose in many fields. With the knowledge that most β-xylosidases are sensitive to the xylose, especially the β-xylosidases from fungi, such as *Arxula adeninivorans*, *Aureobasidium pullulans* and *Trichoderma Reesei* exhibiting a *Ki* for xylose ranging from 2 to 10 mM [21]. However, the β-xylosidases from *Paecilomyces thermophila* and *Scytalidium thermophilum* exhibited a certain tolerance to xylose (Table 3). It was surprising to find that when concentration of xylose was up to 500 mM, it did not decrease the *T. thermarum* β-xylosidase activity, implying a very high tolerance to the inhibition by its hydrolysis product xylose. Thus, the effect of xylose on the Tth xynB3 β-xylosidase activity revealed that the enzyme was not only resistant to the end-product inhibition, but was also activated by xylose at concentrations less than 500 mM. Compared with other β-xylosidases, the *K*_*i*_ for xylose of Tth xynB3 β-xylosidase was higher than that from *Paecilomyces thermophila*, an unnamed bacterium isolated from yak rumen, and other reported β-xylosidases (Table 3).

Moreover, high specific activity for XOs is also demanded for β-xylosidase in enzymatic hydrolysis of hemicellulose. The *V*_*max*_ value of Tth xynB3 β-xylosidase for *p*NPX was 223.3 U/mg, which was approximately 3-fold higher than that of for *p*NPAF. It has been reported that the specific activity of a xylose highly tolerant β-xylosidase from *S. thermophilum* for *p*NPX is 65 U/mg, which is nearly 2-fold less than the Tth xynB3 β-xylosidase [21]. The Tth xynB3 β-xylosidase was discovered to be the only β-xylosidase displayed insensitive to xylose yet had higher specific activity for *p*NPX and *p*NPAF. The *k*_*cat*_/*K*_*m*_ of Tth xynB3 β-xylosidase for *p*NPX was 1173.4 mM^-1^ s^-1^, approximately 400-fold higher than the β-xylosidase from *Bacillus halodurans* and 100-fold higher than the β-xylosidase from the unnamed bacterium [26,27]. Therefore, the Tth xynB3 β-xylosidase exhibited unexceptionable potential for bioconversion.

The cations (1 mM or 10 mM) investigated in this study had various effects on the activity of Tth xynB3 β-xylosidase. As described in Table 2, 1 mM (or 10 mM) concentration of Co^2+^, Zn^2+^, Cu^2+^, Al^3+^ and Ni^2+^ inhibited the enzyme activity significantly, while Ba^2+^ and Mn^2+^ enhanced the enzyme activity. It was interesting to find that the Tth xynB3 β-xylosidase was slightly influenced by Ca^2+^, which distinguished Tth xynB3 β-xylosidase from the other β-xylosidases that Ca^2+^ strongly stimulated the activity [21,28,29]. It was also found that the Tth xynB3 β-xylosidase could also be activated by chemical reagents significantly such as 0.05% tween 60, 0.05% tris and 10 mM EDTA. The capability of resisting these chemical reagents and cations indicated that the Tth xynB3 β-xylosidase could survive in specific conditions as described above. It is known that the longer active life means the less consumption of the enzyme [30]. Therefore, the enzymes with high thermostability are especially demanded in industrial applications such as in the field of bioconvertion. The residual activity of Tth xynB3 β-xylosidase residual activity was more than 50% of its initial activity after being incubated at 75°C-85°C for 2 h, and the enzymatic hydrolysis of XOs exhibited a high activity in a broad temperature range from 75°C to 100°C.

The capability of the β-xylosidase to hydrolyze XOs was investigated by using 5% substrate incubated with the purified enzyme at 75°C. In 100 μL reaction system, the XOs were completely biodegraded to xylose by 0.3 U purified Tth xynB3 β-xylosidase after 3 h (Figure 4), and the total monose xylose concentration reached at 360 mM. However, under this condition, the xylose did not affect enzymatic reaction. Same as the other β-xylosidases, Tth xynB3 β-xylosidase was also active on xylobiose, xylotriose and xylotetraose [31,32]. The results illustrated that the Tth xynB3 β-xylosidase exhibited high ability for converting the XOs into xylose monomers.

## Conclusions

In this study, a novel β-xylosidase, Tth xynB3 β-xylosidase, from *T. thermarum* DSM 5069 was obtained with a few specific features, as well as the high activity of α-arabinosidase. The Phylogenetic analysis showed that Tth xynB3 β-xylosidase had close relationship with the β-xylosidase from hyperthermophile, and was distant with other xylose-tolerant β-xylosidases. Compared with the enzyme properties from other microorganisms, the Tth xynB3 β-xylosidase possessed higher tolerance to xylose, higher efficiency in XOs hydrolysis and higher thermostability. Therefore, this study provides a novel and useful β-xylosidase/α-arabinosidase with combined properties of high thermostability and xylose-tolerance. These characteristics constitute a powerful tool for improving the enzymatic conversion of hemicellulose to xylose through synergetic action.

## Materials and methods

### Bacterial strains, plasmids and growth media

*Thermotoga thermarum* DSM 5069 was purchased from DSMZ (http://www.dsmz.de). It was grown anaerobically at 80°C as described [19]. *Escherichia coli* Top10 and BL21 (DE3) cells were grown at 37°C in Luria-Bertani (LB) medium and supplemented with ampicillin when required. The expression vectors pET-20b (Novagen) were used as cloning and expression vector.

### DNA manipulation

DNA was operated by standard procedures. Plasmid Kit and Gel Extraction Kit (BIOMIGA, Shanghai) were used to purify the plasmids and PCR products. DNA restriction endonucleases and T4 DNA ligase were purchased from TaKaRa (Dalian, China). DNA transformation was carried out by electroporation using Gene Pulser (Bio-Rad, USA).

### Plasmid constructions

The DNA fragment with a size of about 2,300 bp was amplified from *T. thermarum* DSM 5069 genomic DNA with the primers Tth xynB3-1 and Tth xynB3-2 (Table 4). Fragments from the amplified DNA were then digested with *Nde I* and *Xho I* endonuclease and inserted into pET-20b vector at the corresponding sites*,* yielding the plasmid pET-20b-*Tth xynB3*. 

**Table 4:** Nucleotide sequences of the primers used

**Primer**	**Nucleotide sequence**
Tth xynB3-1	5’-GGAATTCCATATGGATCTTTACAAGAATCCAAATGTAC-3’
Tth xynB3-2	5’-CCGCTCGAGCTCGATCTTTGTATTTGTGAAGAAAAC-3’

### Expression and purification

Plasmid pET-20b-*Tth xynB3* was transformed into *E. coli* BL21 (DE3), and induced to express recombinant *Tth xynB3* β-xylosidase by adding isopropyl-β-D-thiogalactopyranoside (IPTG) to final concentration of 0.5 mM at OD_600_ approximately 0.8, and incubated further at 30°C for about 12 h.

200 mL of the recombinant cells carrying pET-20b-*Tth xynB3* were harvested by centrifugation (10,000 g, 15 min, 4°C), and washed twice with distilled water, resuspended in 5 mL of 5 mM imidazole, 0.5 mM NaCl, and 20 mM Tris–HCl buffer (pH 7.9). The cell extracts after sonication were heat treated (70°C, 30 min), and then cooled in an ice bath, and centrifuged (15,000 g, 4°C, 20 min). The obtained supernatants were loaded on to an immobilized metal affinity column (2 mL) (Novagen, USA) with a flow rate 0.2 mL min^-1^. Finally, 1 mL fractions were collected by eluting with 0.4 M imidazole, 0.5 M NaCl, and 20 mM Tris–HCl buffer (pH 7.9). SDS-PAGE was carried out to verify the purity of the target proteins [33], and the protein bands were analyzed using an image analysis system (Bio-Rad, USA). Purified protein concentration was determined by the Bradford method using albumin from bovine serum (BSA) as a standard. Oligomerization state of Tth xynB3 β-xylosidase was determined by size exclusion chromatography on a Ӓ KTA*FPLC*™ (GE Healthcare Life Sciences) system with a Superdex 200 10/30 GL column as described by Zhang et al. [34].

### β-Xylosidase/α-arabinosidase assays

Substrate *p*-nitrophenyl-β-D-xylopyranoside (*p*NPX, Sigma, USA) was used for β-xylosidase activity analysis and *p*-nitrophenyl-α-L-arabionfuranoside (*p*NPAF, Sigma, USA) for α- arabinosidase activity analysis. Under standard assay condition, the purified enzyme (0.1 μg) was incubated with 10 μL of 20 mM substrate pNPX or pNPAF in 50 mM imidole-potassium buffer (pH 6.0) for 20 min at 85°C. The total reaction volume was 0.2 mL. Subsequently, 600 μl of 1 M Na_2_CO_3_ was added to stop the reaction. The *p*-nitrophenol absorbance (*p*NP) was measured at 405 nm. One unit of β-xylosidase or α-L-arabinosidase activity was defined as the amount of enzyme releasing 1 μmol *p*NP per minute. All enzymatic activities shown in figures are average values of three separate determinations.

The optimum pH for β-xylosidase was determined by incubation at **various** pH (pH 4.0-8.5) at 85°C for 20 min in 50 mM imidole-potassium buffer. The optimum temperature for the enzyme activity was determined by standard assay ranging from 60°C to 100°C in 50mM imidole-potassium buffer at pH 6.0. The results were expressed as relative activity to the value obtained at either optimum temperature or optimum pH. Thermostability assays were determined by measuring residual β-xylosidase or α-arabinosidase activity after pre-incubation of enzymes at 75°C, 80°C, 85°C, 90°C and 95°C for 30 min, 60 min, 90 min and 120 min. The activity of the enzyme without pre-incubation was defined as 100%.

The effects of metal ions and chemical reagents on β-xylosidase or α-L-arabinosidase activity of purified enzyme (0. 1 μg) were determined. Mg^2+^, Zn^2+^, Mn^2+^, Ca^2+^, Al^3+^, Ni^2+^, Cu^2+^ and Co^2+^ were assayed at concentrations of 1 mM (or 10 mM) in the reaction mixture. The chemical reagents EDTA (1 mM or 10 mM), Tris (0.05%), Tween 60 (0.05%), and SDS (0.1%) in the 0.2 mL reaction mixture were assayed. The enzyme was incubated with each reagent for 1 h at 85°C before the addition of *p*NPX or *p*NPAF to start the enzyme reaction. The activity of the enzyme without the chemical reagents or metal cations was defined as 100%.

The substrate specificity of the enzyme (0.1 μg) was tested by using following substrate, such as *p*-nitrophenyl-β-D-glucopyranoside (Sigma, USA), *p*-nitrophenyl α-D-glucopyranoside (Sigma, USA) and linear arabinan (Megazyme International Ireland). Kinetic constant of Tth XynB3 β-xylosidase was determined by measuring the initial rates at various *p*NPX or *p*NPAF ending concentrations (100, 125, 150, 175, 200, 250, 275, 300, 325, 350 and 400 μM) under standard reaction conditions. The influence of various xylose concentrations (100, 200, 300, 400, 500, 600, 700, 800, 900, 1000, 1100 and 1200 mM) on the β-xylosidase activity was investigated. The *K*_*i*_ value of xylose was defined as amount of xylose required for inhibiting 50% of the β-xylosidase activity and was present as the averages of three separate determinations.

### Phylogenies analysis of Tth xynB3 β-xylosidase

The potential ORF of *Tth xynB3* was searched using the ORF search tool provided by the National Center for Biotechnology Information (http://www.ncbi.nlm.nih.gov). Other 54 β-xylosidases amino acid sequences searching were implemented with Blast at NCBI and against CAZy database (http://www.cazy.org). The multiple sequence alignment tool Clustal X2 was used for sequences alignment [35]. Sequences were further edited and aligned manually by using Mega 5 for editing [36]. Phylogenetic relationships were deduced using the Neighbor-Joining (NJ) and Maximum-Parsimony (MP) methods as performed in Paup 4.0 for the NJ and MP trees. 1000 bootstrap replicates were used for evaluating the trees’ http://topologicalhttp://structure[37]. The trees generated above were displayed using TREEVIEW 1.6.6 ( http://taxonomy.zoology.gla. ac.uk/rod/ treeview.html).

### Xylooligosaccharides degradation

The sugar xylobiose, xylotriose and xylotetraose that prepared for 10% XOs were purchased from Sigma Chemical Co. The XOs were obtained from the cornstalk according to the method as Rémond et al. described [7]. The XOs was treated with purified Tth xynB3 β-xylosidase, and the degradation was subjected to analysis of thin-layer chromatography (TLC). The reaction mixture (100 μL) contained 5% xylooligosaccharides (wt/vol) and 0.3 U of Tth xynB3 β-xylosidase in 50 mM imidole-potassium buffer (pH 6.0). The reaction was carried out for various times (0.5 h, 1 h, 2 h and 3 h) at 75°C, and stopped by heating for 15 min in a water bath. After centrifuged for 15 min at 12,000 rpm, the supernatants of the reaction mixtures were applied on silica gel TLC plates (G, Qingdao). Sugars on the plates were separated with a solvent system consisting of *n*-butanol, acetic acid, and water (2:1:1, by vol/vol), and detected using the orcinol/concentrated sulfuric acid reagent [38].

## Competing interests

The authors declare that they have no competing interests.

## Authors’ contributions

Hao Shi and Xun Li carried out the cloning and expression and drafted the manuscript. Huaxiang Gu, Yingjuan Huang and Liangliang Wang helped to purified and characterized the Tth xynB3 β-xylosidase. Yu Zhang and Xun Li helped to revise the manuscript. Fei Wang directed the over-all study and revised the manuscript. All authors read and approved the final manuscript.
